# Characteristics of *Haemophilus influenzae* carriage among healthy children in China: A meta-analysis

**DOI:** 10.1097/MD.0000000000035313

**Published:** 2023-11-03

**Authors:** Cui Ma, Yutuo Zhang, Hua Wang

**Affiliations:** a Department of Microbiology, Hebei North University, Zhangjiakou, Hebei Province, China; b Department of Microbiology, Hebei North University, Zhangjiakou, Hebei Province, China; c Hebei North University Library, Zhangjiakou, Hebei Province, China.

**Keywords:** carriage, *Haemophilus influenzae*, healthy children, meta-analysis

## Abstract

**Background:**

*Haemophilus influenzae* (Hi) commonly causes invasive and noninvasive bacterial infections. Nationwide investigation on the carriage characteristics of *H influenzae* in healthy children in China is lacking. We reviewed the prevalence of *H influenzae* infections in this population.

**Methods:**

PubMed, CNKI, Wanfang, VIP, and CBM databases were electronically searched to collect cross-sectional studies on the prevalence of Hi among healthy children in China from inception to November 2021. Two reviewers independently screened the literature, extracted the data, and assessed the risk of bias in the included studies. Meta-analysis was performed using Stata 14.0.

**Results:**

A total of 28 studies involving 14,301 children were included, among whom there were 2878 children with Hi. The pooled carriage rate of Hi was 0.21 (95% CI: 0.17–0.25). Subgroup analysis indicated no significant sex- or age-related differences. The proportion of Hi in winter (29%) was higher than that in other seasons. Results indicated significant differences among the provinces, with carriage proportions ranging from 0.11 to 0.60. The proportion of nontypeable *H influenzae* (NTHi) was higher than that of the capsular type. The proportion of Hib in the capsular type (2%) was higher than that in other serotypes.

**Conclusions:**

The carriage rate of Hi in healthy children in China was 21% with no sex-related age differences. The proportion of Hi in winter was high, and the proportions of Hi in different regions were significantly different. NTHi was the predominant serotype detected in children.

## 1. Introduction

*Haemophilus influenzae* (Hi) is an opportunistic pathogen that colonizes the human nasopharynx. When the immunity of the body decreases or the local microecological environment becomes imbalanced, community-acquired pneumonia and bacterial otitis media develop in children.^[1]^ Hi can also invade the bloodstream and cause sepsis, purulent meningitis, and other invasive diseases,^[2]^ which seriously threaten the health of children. Studies have shown that a high colonization rate of Hi leads to high morbidity.^[3]^ Therefore, focusing on Hi carriage characteristics in healthy children has important implications for the prevention of Hi infections. Currently, most studies on the epidemiology of Hi in Chinese children are limited to a certain region, and there are no large samples or multicenter epidemiological investigations. This study searched relevant literature published at home and abroad on the prevalence of Hi in children in China and analyzed the characteristics of Hi among healthy children in China to provide a basis for child health policy decision-making.

## 2. Materials and methods

### 2.1. Literature search and screening

We searched the PubMed, CNKI, Wanfang, VIP, and CBM databases to collect cross-sectional studies on the prevalence of Hi among healthy children in China from inception to November 2021. The following terms were used: ‘‘*Haemophilus influenzae*,” ‘‘Healthy children,” ‘‘prevalence,” ‘‘carriage,” ‘‘epidemiology,” and ‘‘China.” The references of the included studies were reviewed to ensure complete coverage.

Wang Hua and Ma Cui independently conducted preliminary literature screening, extracted data, and crosschecked the literature. During the literature screening, duplicate studies were eliminated, irrelevant literature was excluded by reading titles and abstracts, and studies meeting the inclusion criteria were extracted and included. If there is no consensus, Zhang Yutuo was consulted to assist in determining whether final inclusion is possible.

### 2.2. Inclusion criteria

Studies were included if they met the following criteria: the study subjects were healthy Chinese children aged 0 to 12 years, specimens were obtained from the nasopharynx or oropharynx, identification of Hi, including isolation culture and PCR were done, studies reported the proportion of carriers of a single Hi serotype only, studies were published in English or Chinese, and the studies were of a journal type of publication and the source journal literature was that of Chinese science and technology paper statistics.

### 2.3. Data extraction and quality assessment

Data extraction and quality assessment were performed independently by 2 authors, and a consensus was reached through group discussion. Data were extracted based on the following characteristics: author, publication year, study period and region, number of participants, and number of participants infected with *H influenzae*. The risk of bias in the selected studies was assessed according to the guidelines adopted by the Agency for Healthcare Research and Quality (AHRQ).^[4]^ The AHRQ comprises 11 items. Each study was scored for bias type according to the entry content: every item of the AHRQ was answered as yes, no, or not reported, and only the answer “yes” scored 1, while “no” and “not reported” scored 0. The total score represents the summary assessment of bias risk (high quality,8–11; moderate risk,4–7; low quality, 0–3).

### 2.4. Statistical analysis

Pooled prevalence estimates with 95% confidence intervals (CI) were obtained using random-effects models to account for inter-study heterogeneity in the study design and sample size. Forest plots were generated to describe the carriage proportion, corresponding to a 95% CI for each study, and overall estimate. The Q test was used to test for heterogeneity, and *I*^2^ statistics were calculated to quantitatively evaluate heterogeneity (low, 25%–50%; moderate, 50%–75%; and high, >75%). To identify the potential sources of heterogeneity, subgroup analyses were conducted based on age, sex, season, and serotype. To assess the stability of the pooled results, a sensitivity analysis was conducted by excluding one study at a time. Funnel plots and Egger tests were used to detect publication bias. To evaluate the stability of the results, sensitivity analyses were conducted by excluding each study. Statistical analyses were performed using STATA 14.0. Statistical significance was set at *P* ≤ .05.

## 3. Results

### 3.1. Search results and characteristics of included studies

**Figure 1.**
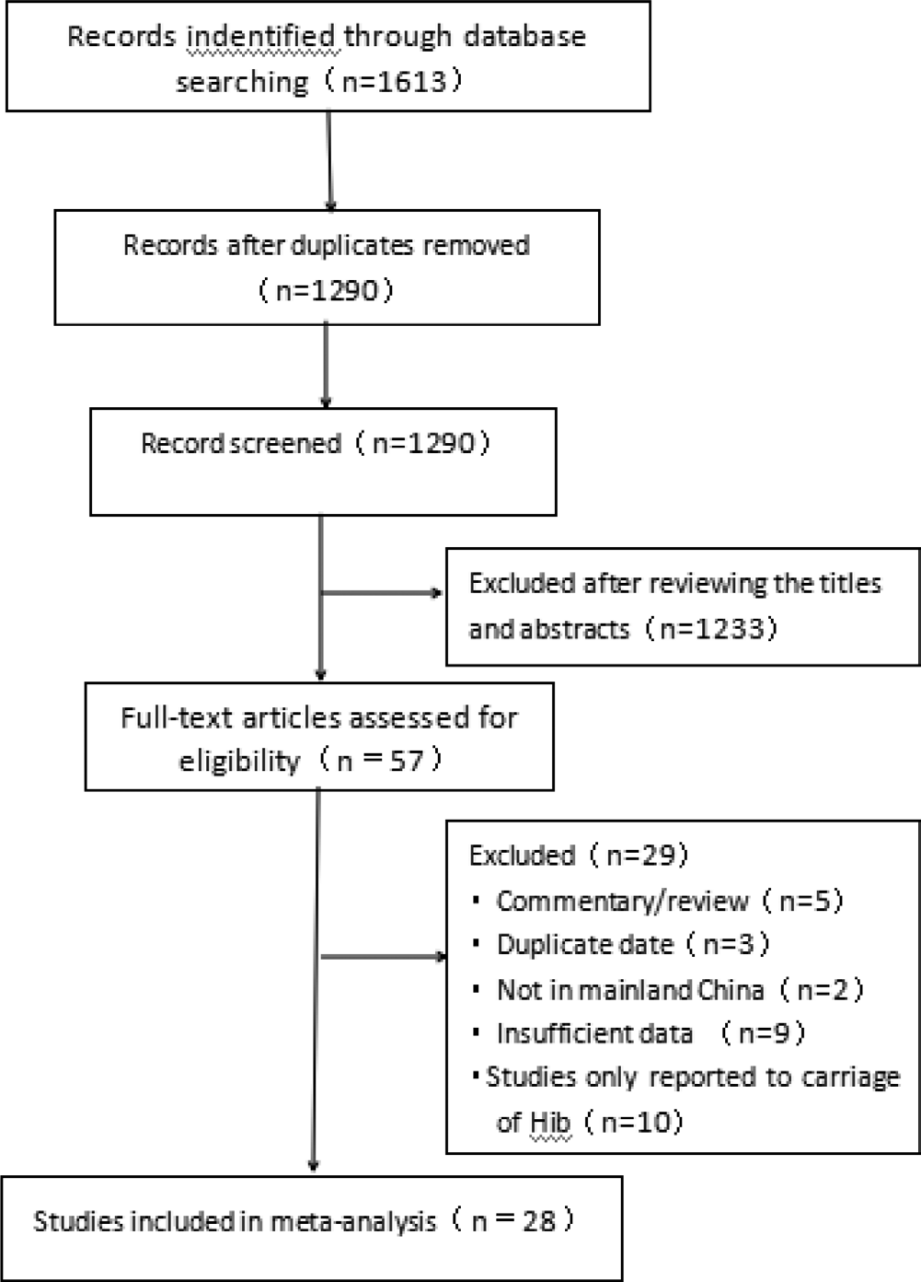
Flow chart of study selection.

We identified 1613 articles from the CNKI (n = 647), Wanfang (n = 330), PubMed (n = 50), Vip (n = 254), and CBM (n = 41332) (Fig. 1). No additional articles were identified in the reference lists of included studies. After removing duplicates and conducting an initial review of titles and abstracts, we assessed the full texts of 57 articles for eligibility of which 28 (28 in Chinese) met the predefined inclusion criteria and were ultimately included in the meta-analysis (Fig. 1).

The characteristics and results of the quality assessment of the included studies are shown in Table 1. All included studies were published between 1993 and 2020, covering 11 provinces nationwide (Table 2).

**Table 1:** The characteristics and results of quality assessment of the included studies.

Study	Period	Location	Participants with Hi	Sample size	Carriage rate (%)	Quality score
Dong FF, 2020^[5]^	2018–2019	Zhengzhou,Henan	106	1000	10.60	7
Zhao DF, 2020	2019	Wuhan, Hubei	175	998	17.74	6
Zhao XJ, 2015	2012–2013	Beijing	45	472	9.53	8
Qiao HX, 2013	2010	Zhangjiakou,Hebei	31	100	31.00	7
Di MZ, 2012	2010	Beijing	73	834	8.75	6
Wei JC, 2011	2008	Dongyang,Zhejiang	13	203	6.40	6
Zhang JM,2010	2009	Quzhou, Zhejiang	12	70	17.14	7
Ye JL, 2009	2007–2008	Zhejiang	43	301	14.29	6
Wang FG, 2009	2007–2008	Hangzhou,Zhejiang	21	101	20.79	5
Meng XJ,2009	2008	Hunan	90	360	25.00	5
Chen DK, 2007	2006	Beijing	134	561	23.89	6
Lai ZG, 2007	2007	Dongguan, Guangdong	109	546	19.96	7
Wu T, 2007	2006	Jiangsu	393	656	59.91	7
Li RC, 2007	2001	Shijiazhuang,Hebei	139	890	15.62	6
Luo XM, 2006	2003	Zhongshan, Guangdong	45	186	24.19	7
Hua CZ, 2005	2002–2003	Hangzhou,Zhejiang	217	848	25.59	9
Zhou H, 2003	2000–2002	Guangzhou,Guangdong	37	150	24.67	6
Zhang YF, 2002	2000	Zhangjiakou,Hebei	56	219	25.57	7
Hou AC, 2002	1998–1999	Beijing	20	307	6.51	6
Zhang HW, 2001	1998–1999	Fuzhou,Guangdong	403	2053	19.63	8
Zhang YT, 2000	1999	Zhangjiakou,Hebei	66	219	30.14	8
Chen LN, 2000	2000	Wuhan, Hubei	6	52	11.54	5
Wang GF,1999	1998	Shanghai	66	255	25.88	7
Lai GX, 1999	1995–1996	Shanghai	49	181	27.07	8
Liu Q, 1993	1993	Beijing	37	115	32.17	6
Chen H, 2009	2009	Shenzhen,Guangdong	45	181	24.86	6
Ye YL, 2012	2011	Shanghai	44	390	11.28	6
Lai GX 2002	1999	Fuzhou, Fujian	403	2053	19.63	9

**Table 2:** Results of subgroup analysis of Hi carriage rates in children.

Subgroup	Literature quantity	Carriage rate [%(95%CI)]	Heterogeneity		Egger test (*P*)
			*P*	*I*^2^(%)	
Gender					
Male	10	25 (17–32)	<.01	69.8	.407
Female	10	27 (19–35)	<.01	78.5	.162
Age					
Infancy	2	24 (10–30)	<.01	99.5	.529
Childhood	5	19 (12–26)	.016	92.6	.164
Preschool age	8	22 (17–28)	<.01	93.7	.197
School age	2	13 (6–21)	<.01	91.2	.004
Region					
Beijing	5	15 (9–22)	<.01	95.6	.15
Hebei	4	25 (17–34)	<.01	90.7	.035
Hubei	1	16 (11–21)	–	–	–
Shanghai	3	21 (10–33)	<.01	93.8	.126
Jiangsu	1	60 (56–64)	–	–	–
Hunan	1	25 (20–30)	–	–	–
Henan	1	11 (9–13)	–	–	–
Fujian	1	20 (18–21)	–	–	–
Zhejinang	3	17 (8–25)	<.01	94.6	.922
Guangdong	7	21 (19–23)	.223	29.8	.013
Season					
Spring	5	13 (8–18)	<.01	87.5	.132
Summer	5	9 (5–13)	<.01	89.3	.493
Autumn	5	16 (9–23)	<.01	93.6	.446
Winter	5	29 (20–38)	<.01	93.5	.844
Type					
Hia	10	0.3 (0.1–0.5)	.186	35.3	.089
Hib	10	2 (0.8–3)	<.01	83.1	.478
Hic	10	0.5 (0.2–0.9)	<.01	95	.159
Hid	10	1 (0.5–2)	.864	0	.721
Hie	10	0.6 (0–1)	<.01	94	.137
Hif	10	0.6 (0.1–1)	<.01	94	.197
NTHi	10	13 (9–17)	<.01	97.8	.784

NTHi = nontypeable *Haemophilus influenzae*.

### 3.2. Overall pooled carriage of *H influenzae* among healthy children

**Figure 2.**
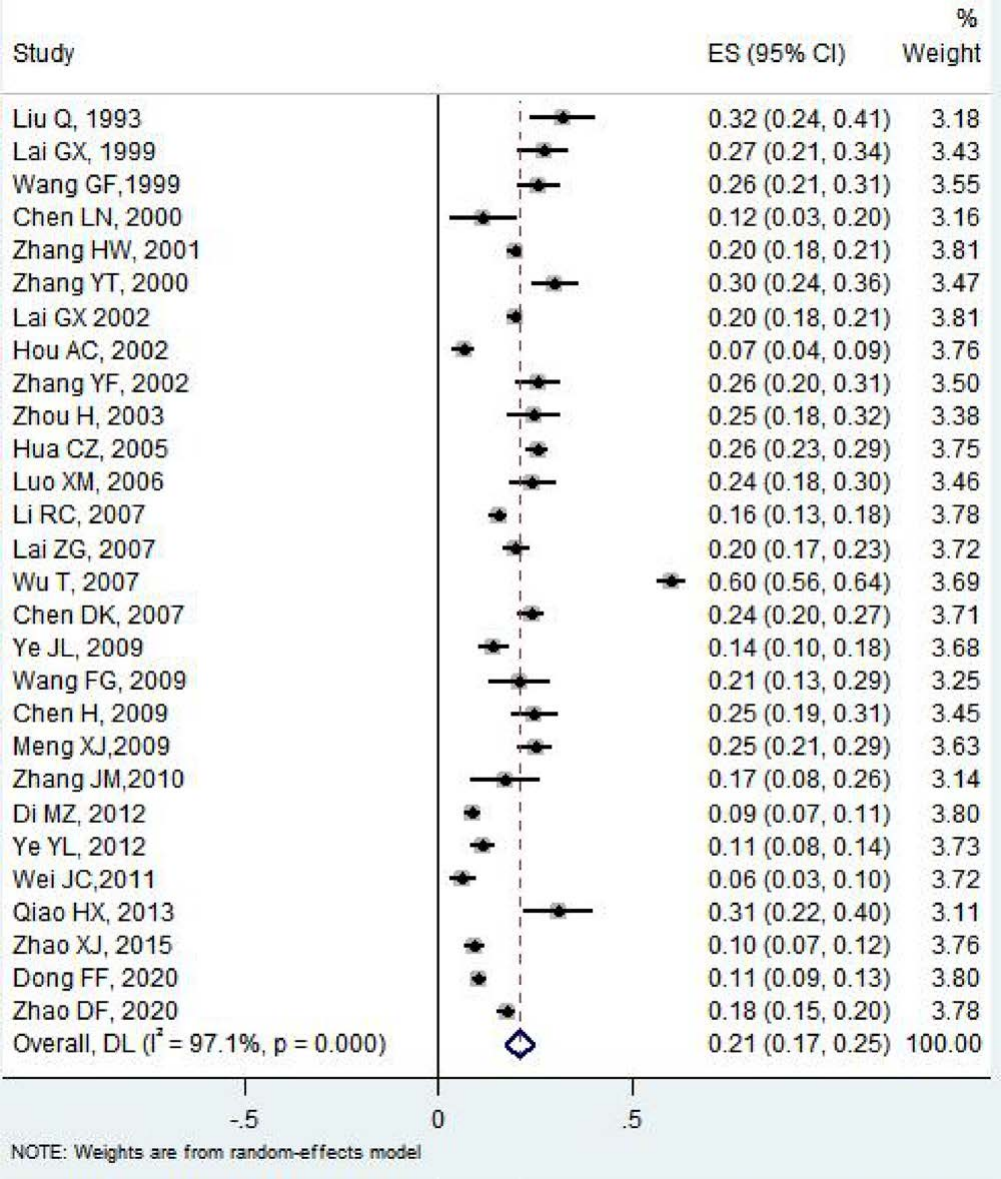
The overall pooled carriage proportion of Hi among healthy children. Hi = Haemophilus influenzae.

A total of 42 studies^[5–32]^ reported the carriage proportion of *H influenzae*, and the overall pooled proportion of carriage was 0.21 (95% CI: 0.17–0.25) with significant between-study heterogeneity (*I*^2^ = 97.1%, *P *< .01) (Fig. 2).

### 3.3. Subgroup analysis

The results of the subgroup analysis of Hi carriage rates in children are shown in Table 2. Ten studies^[7,15–20,24,29,31]^ described the sex-specific carriage proportion of influenzae, and the pooled carriage proportion was 0.25 (0.17–0.32) for males and 0.27 (0.19–0.35) for females, without significant heterogeneity between subgroups (*P* = .71). Among the included studies, 15 studies reported on the prevalence of Hi in children of various ages. Age was divided into infancy (<1 year), early childhood (2–3 years old), preschool age (4–6 years old), and school age (7–12 years old). The pooled carriage proportion was 0.24 (0.04–0.52) in infancy, 0.19 (0.12–0.26) in early childhood, 0.22 (0.17–0.28) in preschool age, and 0.13 (0.6–0.21) in school age, without significant heterogeneity between subgroups (*P* = .290). A total of 28 studies^[5–32]^ reported children Hi carriage rates in different regions, and the results showed that the province with the highest Hi carriage rate had a rate of 60%, the province with the lowest rate had a rate of 11%, and Hi carriage rates varied among children (*P < *.001). Five studies^[5,9,24,29,32]^ described the seasonal carriage proportion of *H influenzae*. The seasons were divided into spring (March–May), summer (June–August), autumn (September–November), and winter (December–February). The prevalence of Hi in children was 29% in winter, which was higher than 13% in spring, 9% in summer, and 16% in autumn (*P* < .001). Ten studies^[9–11,13,14,20,24,27,28,32]^ reported the carriage proportion of specific serotypes. The carriage rate of NTHi was 13%, which was higher than that of the capsular type. The Hib carriage rate in the capsular type was 2%, which was higher than that of the other serotypes.

### 3.4. Sensitivity analysis and publication bias

**Figure 3.**
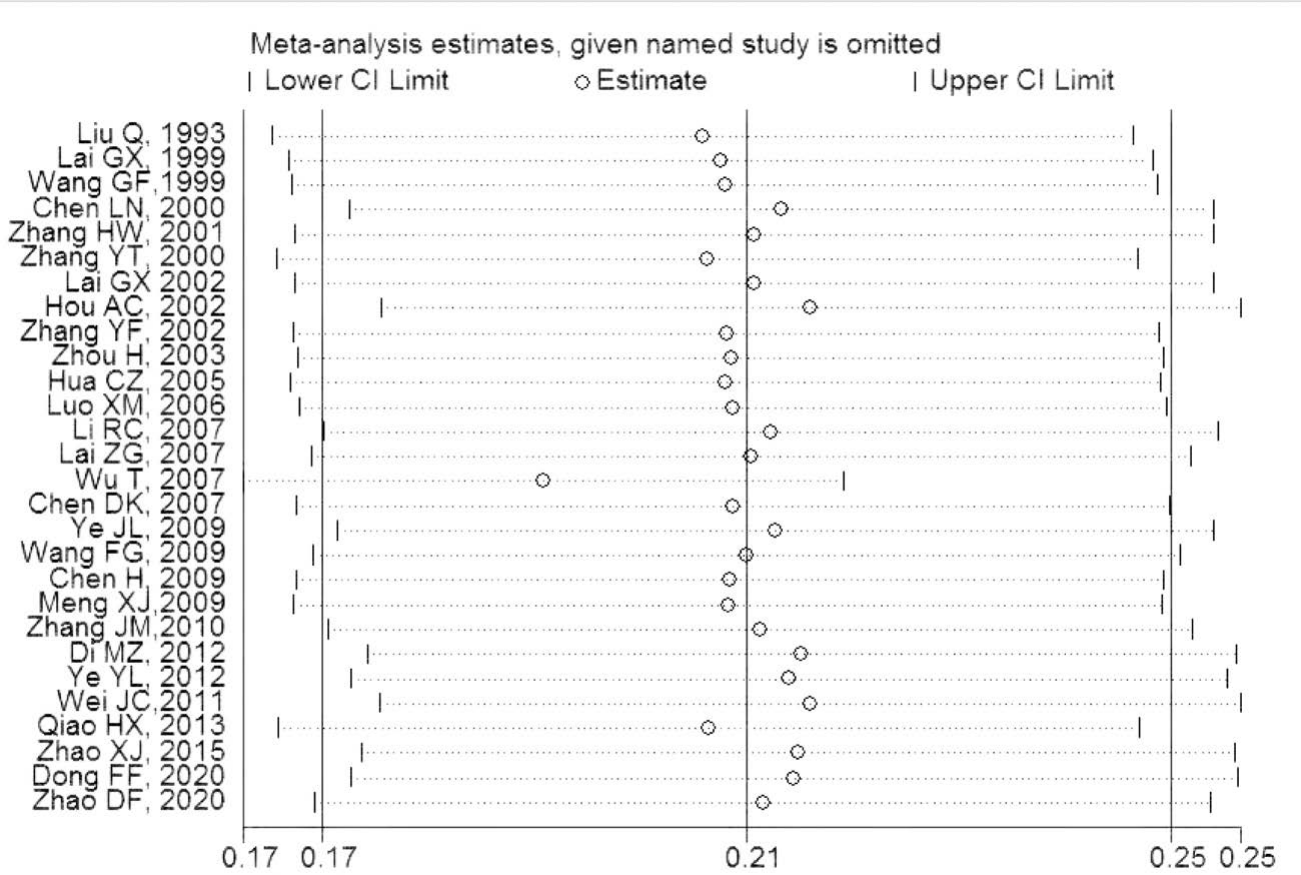
Sensitivity analysis for the effect of individual studies on the pooled carriage proportion.

**Figure 4.**
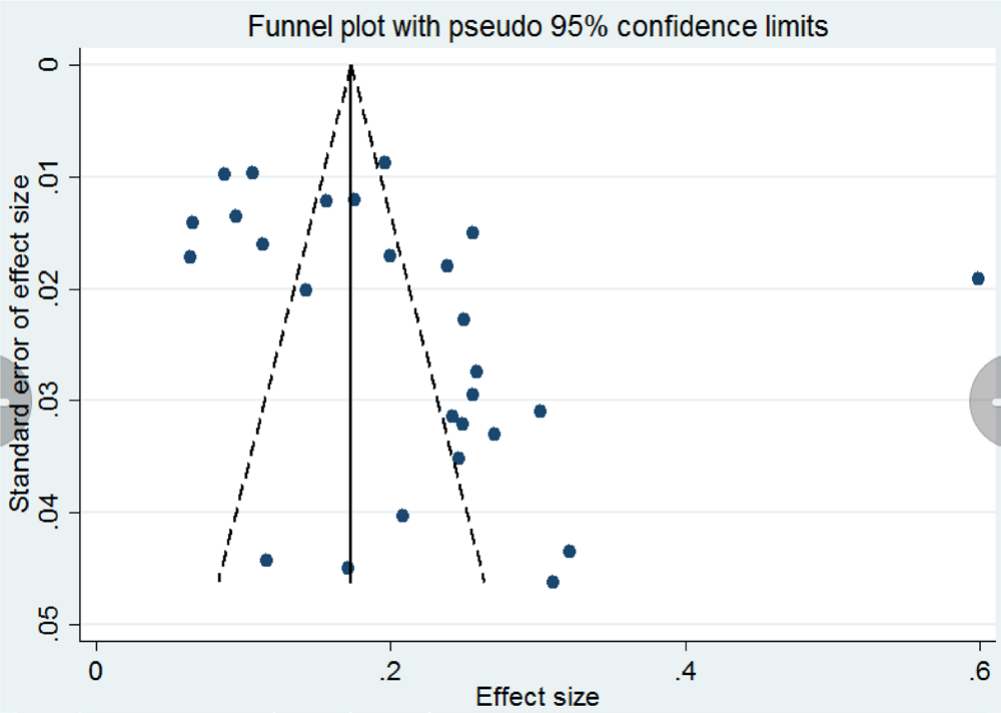
Funnel plot test for publication bias for the pooled prevalence of Hi. Hi = *Haemophilus influenzae*.

**Figure 5.**
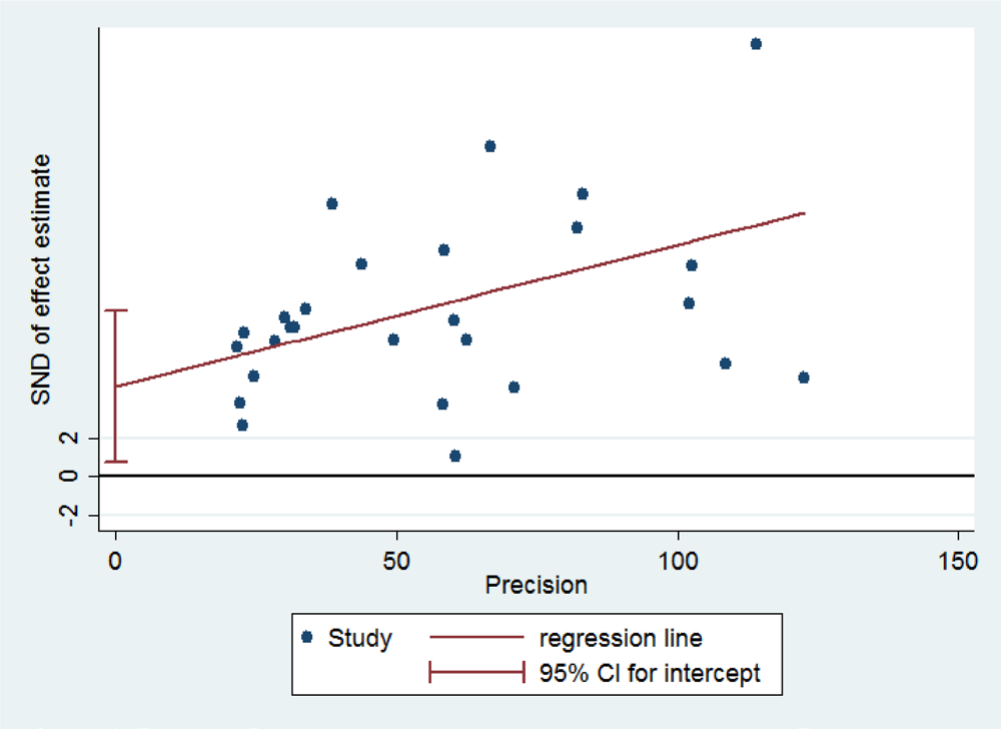
The Egger funnel plot of the 28 included studies in the meta-analysis.

Sensitivity analysis was performed by excluding individual studies to detect changes in the conclusions of the statistical analysis. After excluding each study, the statistical analysis remained unaffected, indicating that the results were stable (Fig. 3). The funnel plots showed a possible publication bias (Fig. 4). Egger test showed no publication bias for studies on children with Hi (t = 2.0, *P* = .054) (Fig. 5).

## 4. Discussion

Hi colonization in the nasopharynx of healthy individuals is closely related to the transmission and occurrence of diseases and the development of immunity. Hib vaccination is an important factor that affects the epidemiological characteristics of Hi. Currently, approximately 98% of the WHO member states and 52% of infants globally receive the Hib vaccine through immunization programs. Since 2014—when Hib was included in Iran routine immunization program—and as of 2019, Hi has been detected in 267 of 902 healthy children in Tehran, Iran, with a carriage rate of 29%.^[33]^ Among 565 healthy children in Rochester, New York, the Hi carriage rate was 5.9% in 2020.^[34]^ Currently, the Hib vaccination is voluntary in China. This study found that the prevalence of Hi among healthy Chinese children was 21%.^[35]^ Influenced by various factors such as geographical environment, climatic conditions, and vaccination, the Hi carriage rate varies significantly in different countries and regions. This study found no sex differences in Hi carriage rates among healthy children, which is consistent with the results of multiple studies conducted in China and abroad. The study population consisted of healthy children aged <12 years. This was because of insufficient age information or inconsistent age groups in the included studies. There was no significant difference in the number of Hi carriers among different age groups.

Colonization is an important step in the development of pathogenic infections. Diseases caused by Hi are more common in children aged 5 years, especially those under 1 year of age. Infants have a high incidence of Hi disease owing to the lack of nasal hair in the nasal cavity, poor ability to remove pathogens, weak nasal mucosa, and rich local blood vessels, which provide favorable colonization conditions for pathogens. This study found that Hi carriages were higher in winter than in other seasons. Winter festivals have a high incidence of Hi-related diseases, and the winter festival has a high incidence of Hi-related diseases because of the vulnerable cold season airway mucosa local immune function, which easily leads to the spread of pathogens and colonization. Combined with the fact that children immune system is still not fully developed and their anti-infection ability is weak, as well as age increases, children contact with the outside world make them increasingly vulnerable to bacteria.^[36]^ Hi carriage varies among the provinces in China, with the eastern region having the highest Hi carriage rate. Moreover, the included studies were mainly concentrated in the eastern provinces of China, lacking data from the west and north, and all included studies were conducted in a certain city with a small sample size, which led to unstable results. Therefore, large-scale, multicenter epidemiological investigations are required. Since Hib-related vaccines became widely available worldwide in 1985, the carriage rate of Hib has decreased sharply. According to 2015 data from the Centers for Disease Control and Prevention, the Hib carriage rate in developed countries ranges from 0% to 1.5%, whereas that in developing countries ranges from 0.5% to 3%.^[37]^ In these countries, Hib carriage rates have decreased in both vaccinated and unvaccinated populations owing to group effects. Simultaneously, the microflora of the upper respiratory tract is systematically altered, with non-type *H influenzae* and NTHi gradually replacing the Hib niche.^[38]^ A study involving 1192 children from 62 nurseries in Brazil found that the prevalence rates of capsular and NTHi were 8.8% and 23.3%, respectively.^[39]^ Among the capsular types, Hif had the highest prevalence (4.7%), followed by Hia (2.0%) and Hib carriage (0.7%). A study by Giufre et al^[40]^ in Italy in 2015 showed that vaccination reduced Hib transmission and almost all Hi isolates in healthy children were of the NTHi type. In some European countries, non-type b *H influenzae* and NTHi are the main causes of invasive infections. Whole-genome sequencing of 247 invasive Hi cases in Norway between 2017 and 2021 revealed that 170 were NTHi (71.8%).^[41]^ Among the capsular types, 30 (43.5 %) were Hif, 18 (26.1 %) were Hib, and 15 (21.7 %) were Hia. This study found that the prevalence of NTHi in healthy Chinese children was 13%. The prevalence of Hib in the capsular type was 2%, followed by Hid (1%). Antibiotic use may affect Hi colonization, and the effects of recent antibiotic use were not excluded from this study. According to WHO data, NTHi have been placed on the priority list of antibiotic-resistant bacteria, and up to 55% of NTHi can produce lactamidases. This may be because NTHi is the main pathogen colonizing the nasopharynx, and antibiotics are often used to control Hi infections. The frequent use of antibiotics makes NTHi more likely to produce drug resistance genes, thus increasing the carriage of NTHi in the nasopharynx and its spread in the community.

The limitations of this study are as follows: Among the 28 included studies, there were differences in Hi detection standards; some were identified by isolation and culture, while others were identified by PCR. The different sensitivities of these methods may affect detection of the Hi carriage rate. The included studies mainly focused on certain cities in certain provinces and lacked data covering the entire country, resulting in inconsistent results. The literature collected in this study was previously published, which may have caused a potential publication bias owing to the lack of unpublished data.

Mastering the characteristics of Hi carriage among healthy children in China provides a basis for formulating objective and scientific preventive measures that are conducive to reducing the harm caused by Hi and the disease burden.

## Acknowledgments

In this section, you can acknowledge any support given which is not covered by the author contribution or funding sections. This may include administrative and technical support, or donations in kind (e.g., materials used for experiments).

## Author contributions

**Conceptualization:** Cui Ma, Hua Wang.

**Data curation:** Cui Ma, Hua Wang.

**Formal analysis:** Cui Ma, Hua Wang.

**Funding acquisition:** Yutuo Zhang.

**Methodology:** Cui Ma.

**Project administration:** Yutuo Zhang.

**Software:** Cui Ma, Hua Wang.

**Writing – original draft:** Cui Ma.

**Writing – review & editing:** Cui Ma.
